# Implementation of Enhanced Recovery After Surgery (ERAS) protocol for elderly patients receiving surgery for intertrochanteric fracture: a propensity score-matched analysis

**DOI:** 10.1186/s13018-021-02599-9

**Published:** 2021-07-28

**Authors:** Wenhao Zhu, Yinjie Yan, Yijin Sun, Zhaoxiang Fan, Niangkang Fang, Yunlu Zhang, Mengchen Yin, Hongbo Wan, Wen Mo, Wei Lu, Xuequn Wu

**Affiliations:** 1 Shanghai University of Traditional Chinese Medicine, Shanghai, China; 2 LongHua Hospital, Shanghai University of Traditional Chinese Medicine, Shanghai, China; 3 Shanghai Jiao Tong University, Affiliated Shanghai Sixth People’s Hospital, Shanghai, China

**Keywords:** Intertrochanteric fracture, Enhanced recovery after surgery, Fast track surgery, Propensity score

## Abstract

**Purpose:**

Enhanced Recovery After Surgery (ERAS) is a multimodal approach to perioperative care that aims to reduce patient perioperative complications, accelerate patient recovery, and improve patient satisfaction by refining and optimizing all perioperative management processes. By comparing two groups of patients before and after the implementation of ERAS for intertrochanteric fracture (ITF) with a matching analysis of propensity score (PS), we aimed to demonstrate that the implementation of ERAS protocol shortens the length of hospital stay (LOS), reduces pain, decreases the incidence of postoperative complications, and promotes functional recovery of the joint.

**Methods:**

We selected 2 periods of 1 year, before (n=98patients) and after ERAS implementation (n=92patients). Data were collected on patient demographics, operative and perioperative details, LOS, VAS score, Harris score, and 30-day complications. ERAS-trained nurses are engaged to support patients at each step of the pre/per/postoperative process, including completing a satisfaction survey, with the help of a mobile app. PS analyses were used for dealing with confounding bias in this retrospective observational study.

**Results:**

After PS matching, the outcomes of 124 well-balanced pairs of patients were compared (conventional vs ERAS). LOS was significantly reduced from 24.3±3.9 to 15.2±2.9 days (*P*<0.001). With the same preoperative VAS scores, we found that patients in the ERAS group had significantly lower postoperative VAS scores than those in the conventional group at days 3 and 7 (*P*<0.001), but the difference was not statistically significant at day 14. patients in the ERAS group had higher Harris scores than those in the conventional group at 1 and 3 months, but the difference was not significant at 6 months. In addition, we found that only one patient in the ERAS group developed complications, while nine patients in the conventional group developed different complications. There was no significant difference concerning the satisfaction survey.

**Conclusion:**

The introduction of ERAS for ITF in our institution has resulted in a significant decrease in LOS, alleviated patient pain, promoted early recovery of patient’s hip function while effectively preventing complications, and obtained patient satisfaction.

## Introduction

Intertrochanteric femoral fractures (ITF) predominates in the elderly, accounting for 45% of all hip fractures, and the incidence of ITF is increasing with the accelerated aging of the population [1, 2]. At the same time, the complications caused by prolonged bed rest in non-operative patients tend to lead to high disability and mortality rates. Survivors are prone to various sequelae, making it a significant public health problem, which, together with the high cost of treatment, makes it a severe impact on both the health of the nation’s citizens and the society's economy [3–5].

Enhanced Recovery After Surgery (ERAS) is one of the critical concepts leading the development of modern surgery in the twenty-first century, which is first proposed by Kehlet in 1997 [6]. Its core component is the integration of perioperative concepts using a series of tools proven effective by evidence-based medicine to intervene in perioperative patients to reduce surgical stress and complications, shorten hospital stays, reduce financial costs, and accelerate postoperative recovery [7, 8]. In recent years, the concept of accelerated rehabilitation surgery has been increasingly applied in orthopedics, and some scholars have confirmed that the application of ERAS has achieved good results in orthopedics, including the clinical application in the field of artificial joint replacement can reduce postoperative hospitalization time and mortality, increase patient satisfaction, and reduce hospitalization costs [9–11]. However, there is little information about the application of the ERAS concept in ITF.

Surgical treatment has excellent advantages in relieving pain, restoring hip function early, improving quality of life, and avoiding complications such as cardiovascular accidents and lower limb venous thrombosis caused by long-term bed rest. Therefore, there is a clinical consensus on the surgical approach for ITF [3, 4, 12]. Currently, PFNA has the advantages of easy operation, more minor trauma, and stable fixation compared with other internal fixation, and is characterized by the addition of BO and the essence of minimally invasive surgery while maintaining the firm fixation and biomechanical stability of AO, which is suitable for all types of ITF and is currently one of the primary surgical modalities for clinical treatment of ITF [4, 13]. Although the rate of disability and death in elderly ITF has been gradually reduced by the continuous improvement of all aspects of perioperative therapy, the high risk of postoperative complications, longer LOS and higher costs, functional worsening, and diminished quality of life are unsatisfactory to both patients and physicians [14]. Therefore, to reduce the occurrence of these problems and allow patients to recover as early as possible in a safe manner, it is not only necessary to regulate the treatment modalities in the perioperative period, but more importantly, to fine-tune each management before, during, and after surgery, and to intervene early or prophylactically to reduce the possibility of adverse outcomes for patients. ERAS is the integration of a series of perioperative treatment modalities proven to be effective by evidence-based medicine. The application of these optimized measures in a generalized manner to reduce the physiological and psychological traumatic stress of patients and achieve the purpose of promoting their recovery. Therefore, the application of ERAS is well suited to the current needs of treating ITF.

At the same time, Chinese medicine treatment, including Chinese herbal medicine, acupuncture, and tendon manipulation, has gradually been proven to improve local tissue blood circulation, prevent lower limb venous thrombosis, promote injured tissue repair, eliminate traumatic sterile inflammation, and enable accelerated fracture healing [15–17]. The addition of Chinese medicine treatment modalities not only adds to the treatment ideas for dealing with various complications in the perioperative period, but also accelerates the patient’s recovery in conjunction with Western medical treatment modalities. Therefore, it is essential to explore how to establish an effective combined Chinese and Western medicine treatment and management strategy under ERAS for the rapid recovery of ITF patients.

Our institution began exploring the implementation of a guided protocol regarding the development of ERAS for ITF in September 2019. We compare patients who did not implement ERAS with those who implemented ERAS treatment, observing and comparing LOS, economic indicators, Harris Hip Score (HHS), VAS score, and complications in both groups, to develop an optimal management plan for the preoperative, intraoperative, and postoperative periods, respectively. PS analysis is very reliable in epidemiological studies that produce slight bias and is particularly useful in surgical studies, where randomization is more difficult [18, 19]. Therefore, in this retrospective observational study, PS analysis was used to deal with confounding bias. One of our purposes is to describe the importance of the ERAS concept in treating ITF, including the advantages in applying ERAS and our reasons for using ERAS in patients with ITF. Another aim is to confirm whether ERAS protocols can shorten LOS, reduce pain, decrease the incidence of postoperative complications, and facilitate functional recovery of the joint. This paper also shows the details of our ERAS protocol for ITF to provide references and ideas for our colleagues in the treatment of ITF.

## Patients and methods

### Inclusion and exclusion criteria

This is a retrospective analysis of prospectively gathered data from our institution registry, which contains the records of all elderly patients who received surgeries for intertrochanteric fracture. The study was approved by the Institutional Ethics Committee.

Inclusion criteria was made according to the PICOS (Patient, Intervention, Comparison, Outcome and Study design) principle. Inclusion criteria were as follows: (1) the patients are over 75 years old, (2) X-ray or CT indicate unstable intertrochanteric fractures, (3) treated with PFNA intramedullary fixation, (4) being willing to undergo surgery with the ERAS pathway, and (5) being willing to give informed consent. Exclusion criteria were as follows: (1) patients are with open fractures or pathological fracture caused by tumor, infection, or tuberculosis; (2) patients are with congenital hip dysplasia or osteonecrosis of the femoral head; (3) hip surgery before enrollment; and (4) failure to understand or sign informed consent. To maintain uniformity, we only included patients who underwent PFNA intramedullary fixation by either open reduction or closed reduction techniques. All the surgeries were performed by two surgeons (WHB and WXQ).

The ERAS protocol was established in our hospital in September 2019. Over the next 3 months, the procedural pitfalls were ironed out using the best available literature and Delphi expert-opinion method—with all the piece of the protocol being consistently applied from December 2019 onwards. The practical details of how each component would be executed were agreed upon in a final meeting, before the ERAS protocol was implemented in totality. A multi-disciplinary team comprised of orthopedic surgeons, physicians, anesthesiologists, physiotherapists, and nurses finalized an ERAS protocol keeping in mind the hospital practices and resources. This led us to selected 2 periods of 1 year, before (Group Conventional, from January 2019 to December 2019, n=98 patients) and after implementing of the ERAS (Group ERAS, from January 2020 to December 2020, n=92 patients).

### ERAS care pathway

The typical pathway of our ERAS protocol begins with patient education in the out-patient department when the option of surgery is offered to the patient. At our center, a 24-h unit with trained nurses is dedicated to the support of ERAS in which a patient debriefing session is held once the intervention is scheduled. After meeting with the anesthesiologist and physiotherapist, an ERAS nurse explains the pre-and postoperative stages of the surgical procedure, the prescribed home medication, potential complications, and hospital stay and evokes the main scenarios that can occur early after discharge. Most importantly, the patients’ expectations are tapered, and realistic goals for recovery are set. A telephonic follow-up is performed 48 h and 1 week after discharge—and the patients have access to the treating team by a 24 × 7 telephone helpline in the event of an emergency.

Patients on the ERAS program are managed by a multidisciplinary team of physicians and nurses. Specialized physicians or nurses provide reasonable explanations in situations in which patients have doubts or resist the relevant treatment to ensure full compliance.

### ERAS procedure

The basic components of the multi-disciplinary ERAS procedure, which we used are shown in Table 1.

**Table 1:** Summary of the ERAS protocol used in the present study

**Preoperative**	Educational program	(1) Understand the patient, assess the condition
		(2) Psychological, nutrition, surgery, rehabilitation education
		(3) Good communication
		(4) Emphasize active function exercise
		(5) Advocate deep breathing, upper limbs pull rings and other cardiopulmonary exercise
	Management of nutrition	(1) If there is hypoalbuminemia and severe anemia, actively look for the original disease and correct it
		(2) When necessary, human serum albumin 10g Ivgtt
		(3) Megaloblyte anemia: folate 5-10mg Po Tid+ vitamin B12 0.5mg Im Tiw
		(4) Iron deficiency anemia: EPO 10,000 IU Ih Tiw+ Ferrous succinate 0.2g Po Tid
	Management of dietary	(1) Eat a high protein diet
		(2) Before anesthesia 6h fast protein liquid (such as milk, broth)
		(3) Before anesthesia 4h fast carbohydrates (such as rice porridge, steamed bread)
		(4) 2h before anesthesia, do not drink clear liquid
		(5) When necessary, 250-500ml glucose was dropped 2-3h before operation
	Management of sleep	(1) Sedative hypnotic or anti-anxiety drugs
	Management of pain	(1) Routine use of anti-inflammatory analgesics such as celecoxib 200mg Po Bid
**Intraoperative**	Selection of anesthesia	(1) General anesthesia (laryngeal mask or endotracheal intubation)
		(2) Combined with local infiltration anesthesia: ropivacaine 200 mg+80 ml saline was injected into the incision and surrounding deep needle
	Control of bleeding	(1) Blood pressure control: systolic blood pressure control in 90-110mmhg
		(2) Bleeding control: 5-10 min before skin incision, tranexamic acid should be dropped 15-20 mg/kg
	Management of body temperature	(1) Monitor and dynamically adjust the operating room temperature, do a good job of keeping warm
		(2) Reduce limb exposure, for patients covered inflatable heating blanket
		(3) The infusion of liquid will be first heated to 37°C
	Prevention of infection	(1) Ensure the operating room environment, control the number of patients involved in the operation
		(2) Strict disinfection towel, as far as possible to shorten the operation time and reduce the surgical trauma, the operation field repeatedly rinse
		(3) Preoperative 0.5-2h intravenous antibiotics
		(4) If the operation time exceeds 3h, or blood loss > 1500ml with the second dose
		(5) The effective coverage time of antibacterial drugs includes the whole surgical process and 4 hours after surgery, and the total prevention time is no more than 24h
**Postoperative**	Management of anesthesia	(1) General anesthesia wake up: drink water before eating
		(2) Moxapride 5mg Po Tid to improve gastrointestinal motility
		(3) Selection of anesthesia
	Management of rehydration	(1) Avoid a large amount of fluid replacement: infusion volume from 25 to 40ml (Kg/d) is appropriate
		(2) Control the infusion speed: the infusion speed of elderly patients is from 100 to 120ml/h is appropriate
		(3) Monitor blood routine, liver function, kidney function and cardiac function indicators
	Management of drainage tube	(1) No drainage or catheter was placed
	Control of nausea and vomiting	(1) Intraoperative intravenous use of dexamethasone 10 mg
		(2) Use ondansetron when necessary
	Management of sleep	(1) Sedative hypnotic or anti-anxiety drugs
	Management of pain	(1) Use of automatic analgesia pump for three days
		(2) Sequential use of anti-inflammatory and analgesic drugs, such as celecoxib 200mg Po Bid (recommended reduction of 50% for liver damage and elderly patients)
	Management of activity	(1) Emphasis on early hip, knee and ankle active flexion and extension function exercise, to increase muscle strength
		(2) Exercise passive joint flexion and extension of hip, knee and ankle joints with the help of the physician and CPM, at least 3 times a day, at least 15 minutes each time
		(3) Asked frequently turn over, clap back
		(4) Acupuncture
		(5) Manipulation

These principles cover preoperative management, intraoperative management, and postoperative management to reduce perioperative complications, ensuring safe patient discharge, and promoting early patient recovery.

Preoperative management includes five aspects: educational program, management of nutrition, management of dietary, management of sleep, and management of pain. Preoperative education includes informing patients about the disease and surgery-related conditions and obtaining good doctor-patient communication. We also emphasize the importance of active functional practices after surgery and advocate cardiopulmonary exercises such as deep breathing and upper limb pulling to improve cardiopulmonary function and prevent complications. Management of nutrition involves treating hypoalbuminemia and anemia using human serum albumin, folate, vitamin B12, EPO, and ferrous succinate for different situations. In terms of dietary management, pre-anesthesia diet management is our priority concern. We developed dietary requirements for 2h, 4h, and 6h before anesthesia to prevent the risk of pulmonary aspiration. Two hundred fifty to 500ml of glucose was dropped 2–3h before the operation as needed, which not only provided the patient with energy but also reduced the chance of postoperative nausea and vomiting. Sedative hypnotic or anti-anxiety drugs are used in patients with preoperative insomnia. In addition, routine use of anti-inflammatory analgesics such as celecoxib is an effective way to relieve patients’ preoperative pain.

Intraoperative management consists of four aspects: selection of anesthesia, control of bleeding, management of body temperature, and prevention of infection. We use a combination of general anesthesia and local infiltration anesthesia as our anesthetic modality. Bleeding control measures include blood pressure control and tranexamic acid administration. Regulating the appropriate temperature in the operating room, reducing limb exposure, and heating infusion fluids to 37°C are essential ways to keep our patients’ body temperatures under control. The major measures to prevent infection consist of compliance with the concept of sterility in the operating room and strict intraoperative aseptic operation, preoperative antibiotic use. It is noteworthy that the effective coverage time of antibacterial drugs includes the whole surgical process and 4 h after surgery, and the total prevention time is no more than 24h.

Postoperative management includes five aspects: management of anesthesia, administration of rehydration, management of drainage tube, control of nausea and vomiting, and management of the activity. To prevent gastrointestinal dysfunction caused by anesthetic drugs, our management measures include general anesthesia wake-up, the use of Moxapride, and the choice of different anesthetic modalities. In the management of rehydration, control the volume and rate of rehydration and monitor the safety of rehydration. To prevent hip infections and the chance of urinary tract infections, no drainage tube or catheter was placed. To alleviate postoperative nausea and vomiting, we use dexamethasone intraoperatively and ondansetron when necessary. Postoperative insomnia patients were given the same medication as preoperative. Patients were all placed on an automatic analgesic pump for 3 days, and if there was continued pain, anti-inflammatory and analgesic medications were administered sequentially. Early mobilization was facilitated by a rehabilitation therapist. Finally, the addition of acupuncture and manipulation can relieve local symptoms and improve joint function.

### Postoperative evaluation parameters

Patient demographics, clinical history, comorbidities, and operative details were noted from the hospital records. The outcome measures for the study were as follows:

#### Length of stay

LOS (length of stay) associated with ITF is a major public health issue due to the aging population. It is the most objective outcome of evaluating the recovery pathway. Furthermore, high LOS would correspond with increases in postoperative complications [20]. So, we choose the LOS at discharge as the primary outcome measure to assess the speed of recovery.

#### The costs

Economic indicators, including major medical expenses, bed expenses, drug expenses, inspection expenses, operation expenses, cost of anesthesia, nursing care, and blood transfusion, are the other primary outcome measurement.

#### The Harris Hip Scores

HHS is a widely used indicator to evaluate hip function. The Harris score comprises 10 questions, 2 questions (ROM and absence of deformity) for the physician physical examination component and 8 questions for the patient-reported outcome component.

#### The VAS scores

VAS is a reliable and valid measurement of pain. It has a horizontal, 100-mm-long line, with “no pain” recorded on the left side (score, 0) and “pain as bad as it could be” on the right side (score, 10).

#### Complications

Infection, deep venous thrombosis, urinary tract infection, respiratory tract infection, pulmonary embolism, cerebral vascular accident, and gastrointestinal and myocardial infarction will be recorded at each visit during treatment.

### Satisfactions

Both patient groups were surveyed by telephone. The questionnaire was structured according to a Likert scale. The items included (1) questions about the quality of the clinical results, (2) the overall health status, (3) the overall assessment of the quality of provided care, (4) the duration of the hospitalization, (6) whether patients would repeat the procedure under these conditions, and (7) whether they would consider it adequate for a relative.

### Statistical analysis

SPSS 20.0 statistical software (SPSS Inc., Chicago, IL) was used. Propensity score-matched analysis (PS analysis) is performed in four steps (PS calculation, construction of pairs, assessment of imbalance, and comparison of outcomes). In the first step, we calculated the PS from logistic regression for each patient. The selection of variables included in the model was guided by clinical expertise and a review of the literature. In the second step, we matched one patient who was treated in the first period and one who was treated in the second with an identical or close PS. The matching procedure was realized without replacement and with a greedy algorithm. The nearest neighbor technique, with a predefined caliper of 0.2 of the SD of the logit of the PS, was used. Patients with no close PS were not kept for the next steps. The second step led a reduction in sample size. In the third step, we assessed the comparability of the two groups. A successful matching procedure is inferred if residual imbalance, measured by standardized difference (*d*), is slight for all baseline characteristics. A value of *d* < 0.1 has been empirically considered as acceptable. In the final step, we compared outcomes between the two groups on the matched samples.

Efficacy and safety analyses will be conducted according to the intention-to-treat principle using the “last observation carried forward” rule. Before randomization, baseline characteristics will be collected as descriptive statistics for each patient, including gender, age, BMI, duration of symptoms, preoperative red blood cell count, and preoperative hemoglobin count. The data analysis of the primary outcome is based on the per-protocol population as a supportive analysis. Mean, standard deviation, median, quartiles, and inter quartiles for continuous variables, and frequency for categorical variables will be calculated. Continuous variable followed the normal distribution will be presented as means with standard deviations (SDs) and calculated by an independent sample. Student’s *t*-test was used; otherwise, the data will be expressed as medians with ranges, and non-parametric tests will be used. Categorical variables will be expressed as number (%) and analyzed by χ^2^ test or Fisher’s exact test. A *P* value of less than 0.05 is defined as statistically significant with 2-sided 90% confidence intervals (CIs). Missing data will be input with the last observed response carried forward for all measures using the “last-value-carried-forward” principle.

## Results

### Results of PS matching

After PS matching, outcomes were compared for 62 well-balanced pairs of patients (conventional and ERAS). The baseline demographic details, clinical characteristics, and relevant operative information of the 124 patients in the study groups were depicted in (Table 2). There were no baseline statistical differences between the conventional group and the ERAS group in age, gender, ASA grade, and other demographic characteristics.

**Table 2:** Patient characteristics of propensity score-matched patient groups

Variables	All patients (***N***= 190)		***d***
	Conventional group (n=98)	ERAS group (*n*=92)	
Age	77.3±8.3	78.1±8.2	0.23
Gender (M)	44	54	0.14
BMI	24.6±3.5	25.2±3.1	0.23
Operative side (L)	47	52	0.54
ASA grade (II/III, cases)	98:8	91:1	0.21
Preoperative Hb	10.2±2.3	9.9±2.1	0.19
Preoperative HHS	48.3±3.1	48.6±2.9	0.33
Operating time (min)	55.6±7.1	52.8±9.1	0.21
Blood loss (ml)	123.5±18.2	120.2±19.5	0.31
**Variables**	**Marched patients (*N*= 124)**		***d***
	Conventional group (n=62)	ERAS group (n=62)	
Age	78.0±5.2	81.2±4.9	
Gender (M)	32	30	
BMI	25.2±3.1	25.4±3.4	0.02
Operative side (L)	34	30	0.01
ASA grade (II/III, cases)	60:2	60:2	0.03
Pre-operative Hb	10.3±2.1	10.1±1.9	**0.16**
Pre-operative HHS	48.5±2.9	48.4±3.0	0.01
Operating time (min)	57.2±7.5	54.2±8.7	0.08
Blood loss (ml)	121.5±20.2	124.5±18.2	0.03

Note: Bold indicate when the results of the comparisons between the two groups were statistically significant; *d*, standardized difference

### Follow-up

This study included a 6-month follow-up period. Outcome assessments will be conducted at baseline, as well as at 3, 7, 14 days, 1 month, 3 months, and 6 months postoperatively. Follow-up was completed in all 124 patients, and there were no missed cases.

### General results

There was no significant difference in the baseline VAS scores and HHS in the two study groups. We noted an improvement in both the study groups compared to their preoperative scores.

#### LOS evaluation

A comparison between the conventional and ERAS group concerning various outcome measures is depicted in Table 3. Patients in the ERAS group had a significantly shorter LOS (15.2±2.9 days) as compared to the conventional group (24.3±3.9 days) (*p*<0.001).

**Table 3:** Mean LOS, VAS scores, and mean HHS

Variables	Conventional group (***n***=62)	ERAS group (***n***=62)	***P***
LOS (days)	24.3±3.9	15.2±2.9	**<0.001**
VAS preoperative	8.3±1.3	8.3±1.4	0.493
POD 3	7.3±1.4	6.4±1.6	**<0.001**
POD 7	5.5±1.6	4.3±2.0	**<0.001**
POD 14	1.9±1.3	2.0±1.5	0.563
HHS at 1 months	10.1±1.9	10.3±2.1	**0.021**
HHS at 3 months	48.4±3.0	48.5±2.9	**0.034**
HHS at 6 months	54.2±8.7	57.2±7.5	0.642

Note: *POD* postoperative day; *HHS* Harris Hip Score

#### Symptom evaluation

In terms of VAS score, there was a significant difference at 3 and 7 days after surgery (7.3±1.4 and 5.5±1.6 in the conventional group, 6.4±1.6 and 4.3±2.0 in the ERAS group). However, the difference in VAS scores between the two groups was not significant on day 14.

#### Functional evaluation

According to the final follow up, there was a significant difference at 1 and 3 months after surgery (10.1±1.9 and 48.4±3.0 in the conventional group, 10.3±2.1 and 48.5±2.9 in the ERAS group). However, this difference in HHS between the two groups was not significant at longer follow-up intervals (6 months).

#### Complications

The summary of postoperative complications in 30 days is depicted in Table 4. There were 9 cases of complications in the conventional group and 1 case in the ERAS group postoperative, which have significant statistical difference. Two cases occurred deep venous thrombosis in the conventional group. Four cases and 1 case occurred urinary tract infection in two groups, respectively. Two cases occurred respiratory tract infection in the conventional group. One case occurred cerebral vascular accident in the conventional group. No deaths, no readmission, and no reoperation occurred in either group.

**Table 4:** Comparison of 30-day complications between the two groups

Variables	Conventional group (***n***=62)	ERAS group (***n***=62)
Infection	0	0
Deep venous thrombosis	2	0
Urinary tract infection	4	1
Respiratory tract infection	2	0
Pulmonary embolism	0	0
Cerebral vascular accident	1	0
Gastrointestinal	0	0
Myocardial infarction	0	0
30-day readmission	0	0
30- to 90-day readmission	0	0
Reoperation	0	0

#### Satisfaction survey

Summary of the satisfaction survey is depicted in Table 5. There was no significant difference in “Overall surgical result,” “Current health status,” “Quality of cares,” “I would redo it,” and “I would advise it to a relative” scores in the two study groups, respectively. However, patients in the ERAS group had significantly better satisfaction with LOS (62) as compared to the conventional group (52) (*p*<0.001).

**Table 5:** Comparison of satisfaction between the two groups

Variables	Conventional group (***n***=62)	ERAS group (***n***=62)
Overall surgical result (satisfied or very satisfied)	58	60
Current health status (satisfied or very satisfied)	54	58
Quality of cares (satisfied or very satisfied)	60	60
Satisfaction about LOS (agree or strongly agree)	52	62
I would redo it (agree or strongly agree)	59	59
I would advise it to a relative (agree or strongly agree)	56	55

## Discussion

ITF mainly occurs in the elderly and is prone to complications such as decubitus ulcers, urinary tract infections, and crush pneumonia, which can cause high disability and mortality rates. Surgical interventions have effectively reduced disability and mortality rates, among them, PFNA is currently the best surgical technique for treating ITF. Although the success of PFNA in terms of functional recovery and fracture healing, in clinical practice, there are many complications. In the study by Hu et al. [21], 123 elderly patients with ITF who underwent PFNA had a total blood loss from the day of admission to postoperative days 1 and 3 of 693.5 ± 359.6 ml and 863.8 ± 429.9 ml, respectively, with corresponding hidden blood loss (HBL) of 86.8% and 89.4%. The risk of perioperative occult blood loss was confirmed for PFNA. By a systematic review and meta-analysis by Leo et al. [22], the incidence of postoperative pain was 50% higher in patients who underwent PFNA surgery than in those who underwent InterTAN. In addition, most elderly ITF patients have various underlying diseases, low immunity and poor nutritional status. Trauma and surgical stimulation not only aggravate the existing disease or induce corresponding cardiovascular complications, but also further worsen the nutritional situation. Pain from trauma and surgery can induce cardiovascular complications and increase the incidence of delirium, which in turn is associated with delayed functional recovery, increased mortality, and poor functional outcomes at 6 months postoperatively [23]. Hypoalbuminemia is an independent risk factor for increased postoperative complication rates and mortality, as well as an independent risk factor for acute kidney injury in patients with ITF [24, 25]. Therefore, ensuring that patients safely survive the perioperative period, reducing adverse postoperative complications, and accelerating patient recovery is a hot topic of discussion among surgeons today.

However, a search of articles revealed that most physicians are currently limited to treating one aspect of the perioperative period for ITF. For example, studies on the effect of perioperative analgesia treatment only, studies on the development of perioperative anemia treatment only, or studies on the impact of promoting postoperative rehabilitation treatment only. Of course, a satisfactory outcome cannot be achieved by optimizing one aspect of perioperative therapy alone, but requires a rational integration of all effective perioperative treatment modalities to achieve the goal. At the same time, our team observed the achievements of ERAS in hip and knee replacements. In the study by Huang et al. [26], 1138 patients who underwent hip arthroplasty underwent ERAS. At an average follow-up of 21.2 months, only 12 patients (1.05%) were readmitted for prosthetic dislocation. None of the patients developed the infection, periprosthetic fracture, and/or prosthetic loosening. ERAS was shown to improve short-term clinical outcomes without increasing orthopedic readmission, reoperation, or mortality. In a study by Jiang et al. [27], ERAS protocols in patients over 65 years of age who underwent total knee replacement surgery were effective in relieving perioperative pain, reducing transfusion rates, decreasing complications and length of hospital stay, and improving recovery of joint function and were safer and more effective relative to the traditional pathway.

Therefore, we started to think about refining the treatment model of ITF under the ERAS concept.

### Program development

With the objective, we thoroughly searched Wan Fang Data, CNKI databases, Vip Journal Integration Platform, Chinese BioMedical databases, PubMed, MEDLINE, EMBASE, Cochrane Library, and ISI web of knowledge, and ERAS pathway integrated Chinese and western medicine was established by Delphi expert-opinion process including ten experts, including preoperative management, intraoperative optimization, and postoperative care and rehabilitation. Ultimately, a multi-disciplinary team comprised of orthopedic surgeons, physicians, anesthesiologists, physiotherapists, and nurses finalized the ERAS protocol, keeping in mind the hospital’s practice and resources, and put it into practice to observe the clinical benefits of the ERAS protocol.

LOS is necessarily related to economic indicators, and observation of LOS indicators can indirectly reflect patients’ recovery and correlate with the incidence of postoperative complications also correlated [20]. The observation of LOS helps us to understand the effectiveness, safety, and cost of the treatment. Pain management is one of the most critical components of the ERAS concept. Postoperative pain not only prolongs LOS, but also decreases the patient’s subjective willingness to engage in early rehabilitation exercises, therefore reducing postoperative joint function [28]. The VAS score is a valid, reliable, and easily applicable study and is often used as a criterion for assessing pain intensity. Therefore, we chose VAS scores to evaluate the improvement of patients’ pain. The postoperative joint-specific function will be measured using HHS, which is widely used to evaluate the joint function of life for intertrochanteric fractures patients [29]. We used HHS to evaluate the recovery of joint function in patients with ITF. In addition, observing 30-day postoperative complications helps us to understand the long-term safety of ERAS protocols. The economic indicators also directly reflect the economic benefits of the treatment program.

This study provides a new perspective on peri-operative treatment modalities for ITF. However, during the course of the study, despite the benefits of the ERAS pathway that have been identified, progress in daily practice has been slow. On the one hand, we found that the implementation of the new ERAS pathway required time for multi-disciplinary interfacing, and on the other hand we found that the replacement of the traditional model of peri-operative care was slow in progress. Our team considered ways in which we wanted to change the current situation. First, it required a change in the internal organization of the hospital to reach a consensus view within the hospital. Secondly, as the application of the ERAS concept in clinical practice is not solely dependent on the orthopedic surgeon, but more on multi-disciplinary cooperation, routine training and education of physicians and nurses, etc. Therefore, rapid and effective implementation requires that multi-disciplinary as well as nursing departments and other departments develop clinical treatment pathways for their respective departments under the ERAS pathway in advance.

### Effectiveness of ERAS implementation

Patients in the ERAS group had a significantly shorter LOS (15.2±2.9 days) as compared to the conventional group (24.3±3.9 days) (*p*<0.001). There was no significant difference in the baseline VAS scores and HHS in the two study groups. We noted an improvement in both the study groups compared to their preoperative scores. In term of VAS score, there was a significant difference at 3 and 7 days after surgery (7.3±1.4 and 5.5±1.6 in the conventional group, 6.4±1.6 and 4.3±2.0 in the ERAS group). In terms of HHS, there was a significant difference at 1 and 3 months after surgery (10.3±2.1 and 48.5±2.9 in the conventional group, 48.4±3.0 and 54.2±8.7 in the ERAS group). However, this difference in VAS scores and HHS between the two groups was not significant at longer follow-up intervals (14 days and 6 months). There were 9 cases of complications in the conventional group and 1 case in the ERAS group postoperative, which have significant statistical difference. Two cases occurred deep venous thrombosis in the conventional group. Four cases and 1 case occurred urinary tract infection in two groups, respectively. Two cases occurred respiratory tract infection in the conventional group. One case occurred cerebral vascular accident in the conventional group. No deaths, no readmission, and no reoperation occurred in either group.

In brief, it was found that the speed and safety of early postoperative recovery were significantly higher in the ERAS group than in the conventional group, indicating that this protocol plays a crucial role in the early recovery of patients with intertrochanteric fractures and provides us with references and ideas for the future management of intertrochanteric fractures.

### Satisfaction of all patients

Shorter LOS is usually associated with lower costs and also indirectly reflects the physical recovery of patients [30, 31]. Therefore, the premise that the physical condition can be recovered quickly while reducing financial expenses will enable patients to gain patient preference. In addition, more patients in the ERAS group were satisfied with their physical condition compared to those in the conventional group.

### Limits

Due to the lack of high-quality RCT trial evidence in this study, we lacked to have quality theoretical guidance, and in clinical practice, many decisions have to be made without high-quality evidence. Therefore, we hope that more patients will be included in the trial under the developed ERAS protocol to future confirm the effectiveness of the protocol. In addition, economic indicators are an essential aspect of evaluating clinical trials. Due to the small number of cases in our trial, the difference between implementing the ERAS protocol and not implementing it is not well analyzed, which also requires us to analyze data from a large sample to be able to obtain a meaningful statistical analysis.

## Conclusion

This study presents the feasibility of ERAS application in ITF and confirms the benefits of ERAS in ITF. We believe that ERAS is currently the best concept to optimize the perioperative treatment of ITF.

## Data Availability

The datasets used and/or analyzed during the current study are available from the corresponding author on reasonable request.

## Abbreviations

ERAS: Enhanced Recovery after Surgery
LOS: Length of stay
PFNA: Proximal femoral nail anti-rotation
PS analysis: Propensity score-matched analysis
